# Quantifying the Transmission of Foot-and-Mouth Disease Virus in Cattle via a Contaminated Environment

**DOI:** 10.1128/mBio.00381-20

**Published:** 2020-08-04

**Authors:** Claire Colenutt, Emma Brown, Noel Nelson, David J. Paton, Phaedra Eblé, Aldo Dekker, José L. Gonzales, Simon Gubbins

**Affiliations:** a The Pirbright Institute, Pirbright, Surrey, United Kingdom; b The Met Office, Exeter, Devon, United Kingdom; c Wageningen BioVeterinary Research, Lelystad, The Netherlands; Columbia University College of Physicians & Surgeons

**Keywords:** biosecurity, environmental microbiology, foot-and-mouth disease, foot-and-mouth disease virus, indirect transmission, viral decay, virus survival

## Abstract

Indirect transmission via a contaminated environment can occur for a number of pathogens, even those typically thought of as being directly transmitted, such as influenza virus, norovirus, bovine tuberculosis, or foot-and-mouth disease virus (FMDV). Indirect transmission facilitates spread from multiple sources beyond the infectious host, complicating the epidemiology and control of these diseases. This study carried out a series of transmission experiments to determine the dose-response relationship between environmental contamination and transmission of FMDV in cattle from measurements of viral shedding and rates of environmental contamination and survival. Seven out of ten indirect exposures resulted in successful transmission. The basic reproduction number for environmental transmission of FMDV in this experimental setting was estimated at 1.65, indicating that environmental transmission alone could sustain an outbreak. Importantly, detection of virus in the environment prior to the appearance of clinical signs in infected cattle and successful transmission from these environments highlights there is a risk of environmental transmission even before foot-and-mouth disease (FMD) is clinically apparent in cattle. Estimated viral decay rates suggest that FMDV remained viable in this environment for up to 14 days, emphasizing the requirement for stringent biosecurity procedures following outbreaks of FMD and the design of control measures that reflect the biology of a pathogen.

## Abstract (precis)

Effective control of a disease relies on comprehensive understanding of how transmission occurs, in order to design and apply effective control measures. Foot-and-mouth disease virus (FMDV) is primarily spread by direct contact between infected and naive individuals, although the high levels of virus shed by infected animals mean that virus can also be spread through contact with contaminated environments. Using a series of transmission experiments, we demonstrate that environmental transmission alone would be sufficient to sustain an outbreak. Key observations include that a risk of transmission exists before clinical signs of foot-and-mouth disease (FMD) are apparent in cattle and that survival of virus in the environment extends the transmission risk period. This study highlights the role a contaminated environment can play in the transmission of FMDV and presents approaches that can also be applied to study the transmission of other pathogens that are able to survive in the environment.

## INTRODUCTION

Failure to account for all pathways involved in disease spread results in a superficial understanding of transmission (1, 2), limiting the effectiveness of control measures and our ability to anticipate how an outbreak could progress. Indirect transmission through contaminated environments can contribute to the epidemiology of diseases primarily considered to be transmitted by direct contact, such as those caused by noroviruses (3), avian influenza (AIV) (4), bovine tuberculosis (bTB) (5), and foot-and-mouth disease (FMD) (6). In this context, the term “environment” extends to any area that has housed or had contact with an infected individual that is shedding a pathogen. Where pathogens remain viable, environmental contamination facilitates a complex system of spread in which new infections can occur from multiple sources and occasions beyond contact with the infectious host.

FMDV infects cloven-hooved livestock and wildlife species and is an important pathogen on economic and animal welfare grounds (7). It is spread primarily through direct contact between infected and naive animals (8). However, when control measures to prevent direct contact, such as restrictions on animal movement and culling are imposed, outbreaks can still continue (9). This sustained spread of the virus can involve transmission modes such as indirect contact via fomites and long-distance transport of aerosols. FMDV is present in all excretions and secretions from acutely infected animals (10), so environments are readily contaminated. FMDV has been demonstrated to survive outside the host under various conditions (11–14), enabling infectious virus to remain viable in an environment beyond the period in which an animal would potentially be infectious. Contaminated environments are acknowledged as a risk factor for FMDV outbreaks (15), but only very limited experimental work has been carried out to quantify the role of the environment in transmission (6).

Quantifying emissions from hosts, the levels of contamination in environments, the survival of pathogens in specific environments, and the dose-response relationship between these variables and onward transmission is essential for understanding the importance of environmental contamination and the role it plays in the epidemiology of a disease. In this study, we use a series of experiments to quantify the transmission of FMDV in cattle via a contaminated environment. Pairs of naive calves were exposed for 24 h to environments (rooms) that had previously housed FMDV-infected calves and were subsequently monitored for clinical signs of foot-and-mouth disease (FMD). In addition to challenge outcome, we also measured levels of virus in the animals used to contaminate the environment and in various samples taken from the contaminated environment. The objective of the study was to investigate environmental transmission in greater detail by linking viral shedding, the dynamics of virus detection in the environment, and the dose-response relationship for environmental transmission.

## RESULTS

### Outcome of transmission experiments.

Transmission of FMDV occurred in seven out of the 10 environmental exposures and was observed for environments contaminated by needle-inoculated cattle, by contact-challenged cattle prior to the onset of clinical signs, or by contact-challenged cattle after the onset of clinical signs (Table 1). The timing of infections and virus detection (Fig. S1 in the supplemental material) supports environmental exposure being the route of transmission, with additional evidence from sequencing data for experiment 2 (Fig. S2). The time at which calves exposed to contaminated environments showed clinical signs varied (median: 5 days post exposure [dpe]; range: 2 to 10 dpe), but there was no obvious relationship with how the environment was contaminated (Table 1). FMDV (both live virus and viral RNA) was detected in the calves which developed clinical disease, but was either not detected or detected at very low levels in the calves which did not show signs of disease (Fig. S1). This was particularly the case for those environmental exposures where neither calf developed FMD.

**TABLE 1:** Challenge outcome following a 24-h exposure period of pairs of cattle to an FMDV-contaminated environment

Expt no.	Outcome by source of environmental contamination^*b*^			
	Needle-inoculated cattle (room 1)^*a*^	Contact-infected cattle (room 2)^*c*^	Contact-infected cattle before clinical onset (room 2)	Contact-infected cattle after clinical onset (room 3)^*c*^
1	One animal developed clinical signs 5 dpe	No clinical signs observed in either animal	-	-
2	Both animals developed clinical signs 5 dpe	One animal developed clinical signs 4 dpe	-	-
3	No clinical signs observed in either animal	-	One animal developed clinical signs 6 dpe	One animal developed clinical signs 10 dpe
4	No clinical signs observed in either animal	-	One animal developed clinical signs 2 dpe	One animal developed clinical signs 2 dpe

a In experiments 3 and 4 there was a one-day gap between infected animals being removed from the room and the environmental challenge.

b dpe, days post exposure; -, not applicable.

c Contact-challenged cattle were housed in the room until 3 days after clinical onset.

10.1128/mBio.00381-20.2 FIG S1 Quantification of foot-and-mouth disease virus and viral RNA in cattle used in four environmental transmission experiments. Each plot shows viral titres (log_10_ PFU/ml) in nasal swabs (first row) or oral swabs (second row) or levels of viral RNA (log_10_ tissue culture ID_50_ equivalents/ml) in serum (third row), nasal swabs (fourth row), or oral swabs (fifth row). Samples were taken from needle-inoculated (red symbols), contact-challenged (blue symbols), and environmental-challenged animals. Environmental challenge was by exposure to an environment contaminated by two needle-inoculated animals (black symbols), two contact-challenged animals (magenta symbols, experiments 1 and 2), two contact-challenged animals prior to the onset of clinical signs (magenta symbols, experiments 3 and 4), or two contact-challenged animals from the onset of clinical signs (cyan symbols, experiments 3 and 4). Clinical outcome of challenge is indicated by the symbols, which are filled if clinical signs were not observed in an animal and empty if they were. Download FIG S1, TIF file, 0.6 MB. Copyright © 2020 Colenutt et al. 2020 Colenutt et al. This content is distributed under the terms of the Creative Commons Attribution 4.0 International license.

10.1128/mBio.00381-20.3 FIG S2 Transmission network reconstructed from statistical parsimony analyses of the foot-and-mouth disease viruses isolated from animals in experiment 2. Each node represents a single sequence and nodes are color coded according to the challenge method. Edge width is proportional to the number of mutations accumulated within the transmission chain, with thin lines between nodes indicating no mutations and thick lines indicating between 1 and 4 mutations. Calves 9100 and 9101 were needle inoculated and used as donors in the contact challenge of calves 9102 and 9103. Calf 9104 was infected through the environment contaminated by calves 9100 and 9101, while calves 9106 and 9107 were infected through the environment contaminated by calves 9102 and 9103. Download FIG S2, TIF file, 0.3 MB. Copyright © 2020 Colenutt et al. 2020 Colenutt et al. This content is distributed under the terms of the Creative Commons Attribution 4.0 International license.

### Quantifying environmental transmission.

To quantify the environmental transmission of FMDV, we considered three linked components: (i) virus shedding by the animals used to contaminate the environment; (ii) the dynamics of environmental contamination and virus survival; and (iii) the dose-response relationship for environmental transmission.

**FIG 1:**
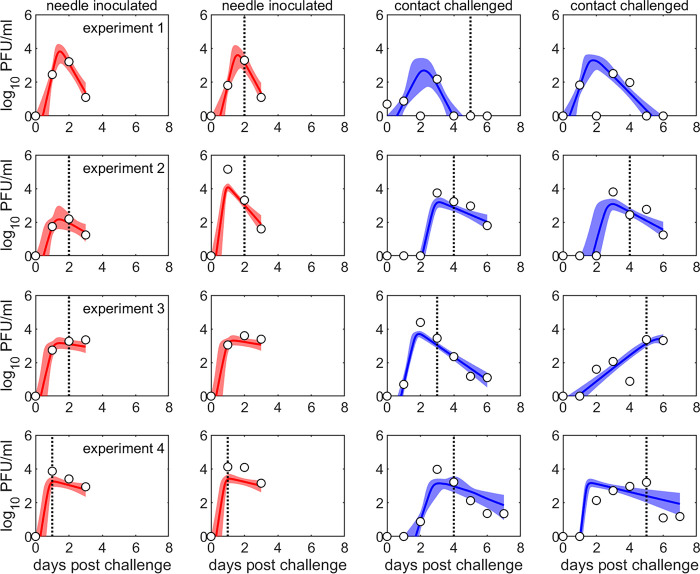
Viral shedding from cattle infected with foot-and-mouth disease virus. Each plot shows the observed (open black circles) and predicted (posterior median [colored line] and 2.5th and 97.5th percentiles [shading]) level of shedding (log_10_ PFU/ml) in nasal and oral swabs from cattle used to contaminate the environment in four transmission experiments (rows). The route of challenge for each animal is indicated by color: needle-inoculated (red) and contact challenged (blue). The vertical dotted line indicates the time of clinical onset, if the animal displayed clinical signs.

**Virus shedding.** Levels of oronasal virus shedding (measured by the total amount of FMDV detected in oral and nasal swabs; used as a proxy for overall shedding by an animal) varied among cattle (Fig. 1; see also Fig. S1). The level of peak shedding did not differ greatly between needle-inoculated cattle (range: 10^2.2^ to 10^5.2^ PFU/ml) and contact-challenged cattle (range: 10^2.2^ to 10^4.4^ PFU/ml) (Fig. 1; Fig. S3). However, the time of peak shedding was earlier and more consistent in needle-inoculated (posterior median for individual animals: 0.8 to 1.5 days) compared with contact-exposed cattle (posterior median for individual animals: 1.4 to 5.6 days) (Fig. 1; Fig. S3).

10.1128/mBio.00381-20.4 FIG S3 Viral shedding parameters for cattle infected with foot-and-mouth disease virus: (A) peak level of virus shed (*V_p_*; PFU/ml); (B) time of peak shedding (*t_p_*; days post challenge); (C) rate for the exponential growth phase (*λ_g_*); and (D) rate for the exponential decay phase (*λ_d_*). Violin plots show the posterior density (shape), the posterior median (black circles) and the interquartile range (black line) for the parameter. Shapes are colored red for needle-inoculated cattle and blue for contact-challenged cattle. Download FIG S3, TIF file, 0.2 MB. Copyright © 2020 Colenutt et al. 2020 Colenutt et al. This content is distributed under the terms of the Creative Commons Attribution 4.0 International license.

**FIG 2:**
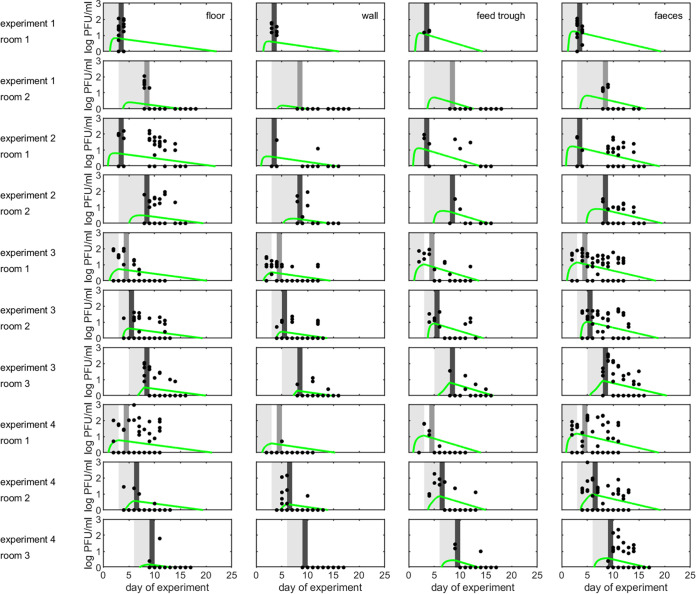
Dynamics of foot-and-mouth disease virus (FMDV) in a contaminated environment. Each plot shows the observed and predicted level of virus (log_10_ PFU/ml) in different rooms (rows) (see Table 1) and environmental samples as follows: floor swabs (first column); wall swabs (second column); feed trough swabs (third column); and feces (fourth column). Observed levels are shown as black points, while predicted levels are shown as the posterior median (green line). For each room light gray shading indicates when FMDV-infected animals were present, medium gray shading indicates when the environmental challenge took place and transmission did not occur, and dark gray shading indicates when the environmental challenge took place and transmission occurred.

**Environmental contamination and virus survival.** The dynamics of FMDV in the environment (Fig. 2) can be described by linking the amount of virus shed by the animals contaminating the environment, the rate at which each sample type becomes contaminated, and the rate at which virus decays in the samples. Consequently, the titer in samples taken from environments contaminated by needle-inoculated animals (room 1 in each experiment) typically increased faster than those taken from contact-challenged animals (rooms 2 or 3 in each experiment) (Fig. 2). The rate at which titers increased differed among sample types (Fig. 2; Table S1), with the highest contamination rate seen in feces and samples taken from the feed trough and the lowest rate for floor and wall samples (Table S2; Fig. S4). The decay rate (Fig. 2) and, hence, half-life of virus, also differed among sample types (Table S1; Fig. S4). The longest half-life (posterior median; 95% credible interval [CrI]) was for floor (7.1 days; 5.1 to 11.8 days) and wall (6.5 days; 4.6 to 11.5 days) samples, while the shortest was for samples taken from the feed trough (3.2 days; 2.4 to 4.6 days) and for feces (4.1 days; 3.3 to 5.3 days) (Table S2).

10.1128/mBio.00381-20.5 FIG S4 Parameters for the dynamics of foot-and-mouth disease virus (FMDV) in the environment: (A) contamination rate (*α*); (B) decay rate (δ); and (C) half-life (in days) for FMDV in different environmental samples. Violin plots show the posterior density (shape), the posterior median (black circles), and the interquartile range (black line) for the parameter. Download FIG S4, TIF file, 0.1 MB. Copyright © 2020 Colenutt et al. 2020 Colenutt et al. This content is distributed under the terms of the Creative Commons Attribution 4.0 International license.

10.1128/mBio.00381-20.8 TABLE S1 Comparison of contamination and decay rates for foot-and-mouth disease virus amongst sample types. Download Table S1, DOCX file, 0.01 MB. Copyright © 2020 Colenutt et al. 2020 Colenutt et al. This content is distributed under the terms of the Creative Commons Attribution 4.0 International license.

10.1128/mBio.00381-20.9 TABLE S2 Estimates for environmental transmission parameters for foot-and-mouth disease virus. Download Table S2, DOCX file, 0.01 MB. Copyright © 2020 Colenutt et al. 2020 Colenutt et al. This content is distributed under the terms of the Creative Commons Attribution 4.0 International license.

Viral RNA was also detected in all environmental sample types and, indeed, at higher levels than infectious FMDV (Fig. S5; cf. Fig. 2). In addition, the half-life for viral RNA was significantly longer (posterior median: 10.6 days; 95% CrI: 8.5 to 13.8 days) compared with infectious virus (Table S2) and did not vary among sample types (Table S1).

10.1128/mBio.00381-20.6 FIG S5 Quantification of foot-and mouth disease viral RNA in environments contaminated by foot-and-mouth disease virus-infected animals. Each plot shows levels of viral RNA (log_10_ tissue culture ID_50_ equivalents/ml) in swabs taken from the floor (first column), walls (second column), feed trough (third column), or in feces (fourth column). Environments were contaminated by housing two needle-inoculated animals (red symbols, all experiments), two contact-challenged animals (blue symbols, experiments 1 and 2), two contact-challenged animals prior to the onset of clinical signs (blue symbols, experiments 3 and 4), or two contact-challenged animals from the onset of clinical signs (black symbols, experiments 3 and 4). Lines show the fitted exponential decay curves (posterior median). Download FIG S5, TIF file, 0.2 MB. Copyright © 2020 Colenutt et al. 2020 Colenutt et al. This content is distributed under the terms of the Creative Commons Attribution 4.0 International license.

**FIG 3:**
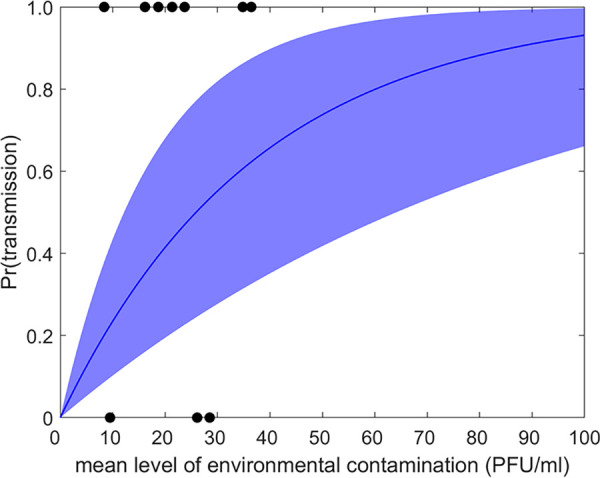
Dose-response relationship for environmental challenge with foot-and-mouth disease virus. The plot shows the posterior median (blue line) and 2.5th and 97.5th percentiles (blue shading). Black circles indicate the inferred mean level of contamination and the outcome (zero, unsuccessful; one, successful) for the 10 environmental challenges conducted in the present study.

**Dose-response relationship for environmental transmission.** An exponential dose-response relationship, in which the dose reflects the level of contamination in the environment and the duration of exposure, adequately captured the probability of transmission (Fig. 3). The estimated dose-response parameter (*β*) was 0.027 PFU^−1^ (95% CrI: 0.011 to 0.057) (Table S2).

### Basic reproduction number for environmental transmission.

The basic reproduction number (denoted by *R*_0_) is “the average number of secondary cases caused by an average primary case in an entirely susceptible population” (16). Based on the above three components (virus shedding, environmental contamination and survival, and the dose-response relationship), we estimated *R*_0_ for environmental transmission of FMDV in our experimental setting to be 1.65 (95% CrI: 0.52 to 4.49) (Table S2). For comparison, *R*_0_ was also calculated based on the attack rate (i.e., 7 out of 10 challenges resulting in successful transmission), which gives an estimate for *R*_0_ of 1.72 (95% confidence interval [CI]: 1.29 to 4.20) (Table S2).

### Implications for control.

**FIG 4:**
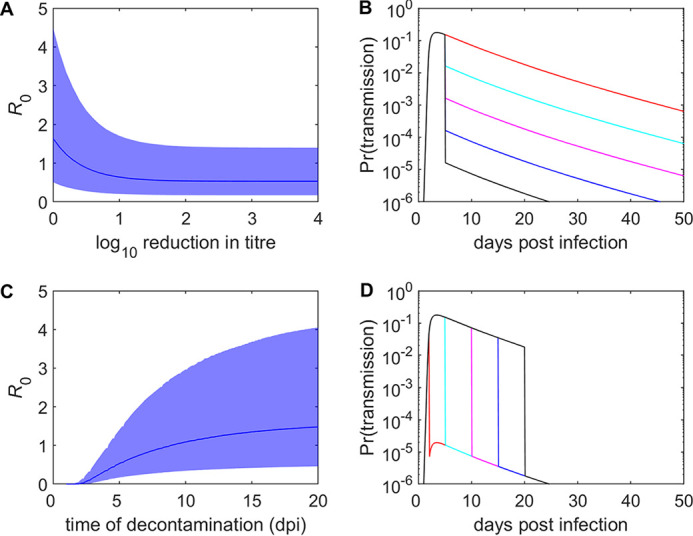
Impact of decontamination on environmental transmission of foot-and-mouth disease virus. (A and B) Basic reproduction number (*R*_0_) (A) and probability of transmission (B) when decontamination is applied at 5 days postinfection (dpi) of the contaminating animal and reduces the viral titer in the environment by different amounts, as indicated in panel B by line color as follows: no reduction (red), 1 log_10_ (cyan), 2 log_10_ (magenta), 3 log_10_ (blue), or 4 log_10_ (black). (C and D) Basic reproduction number (*R*_0_) (C) and probability of transmission (D) when decontamination is applied at different dpi, as indicated in panel D by line color as follows: 2 dpi (red), 5 dpi (cyan), 10 dpi (magenta), 15 dpi (blue), or 20 dpi (black), and reduces the viral titer by 4 log_10_. Panels A and C show the posterior median (line) and 95% credible interval (shading), while panels B and D show the posterior median.

The implications of the results for disease control were assessed by examining the impact of different levels of decontamination and timing of decontamination on *R*_0_ and the probability of transmission (Fig. 4). Increasing the level of decontamination decreases both *R*_0_ (Fig. 4A) and the probability of transmission (Fig. 4B), with a 10-fold reduction in viral titer resulting in a 10-fold reduction in the probability of transmission. However, the reduction in *R*_0_ is limited (Fig. 4A), which reflects transmission prior to decontamination being applied (Fig. 4B). Applying decontamination at earlier times postinfection results in a greater reduction in both *R*_0_ (Fig. 4C) and the probability of transmission (Fig. 4D).

### Detection of FMDV in air samples.

In addition to taking environmental samples, air samples were taken during the course of the experiments. Viral RNA was detected in air samples taken during the environmental challenges (Fig. S6). It was also detected in samples taken at other times, including in rooms housing infected animals prior to the onset of clinical disease (Fig. S6). Live virus was also detected in air samples, although at a lower frequency than viral RNA (Fig. S6).

10.1128/mBio.00381-20.7 FIG S6 Detection of foot-and-mouth disease virus (first and second columns) and viral RNA (third and fourth columns) in ambient air samples taken during transmission experiments. The first and third columns show results for samples taken during the environmental challenges (timing indicated by the gray bars and room by the number), while the second and fourth columns show results for samples taken at other times. Environments were contaminated by either two needle-inoculated animals (red symbols), two contact-challenged animals (blue symbols, experiments 1 and 2), two contact-challenged animals prior to the onset of clinical signs (blue symbols, experiments 3 and 4), or two contact-challenged animals after the onset of clinical signs (black symbols, experiments 3 and 4). When the air sample was taken, the room was contaminated and empty (open circles); the room was contaminated and contained naïve animals (closed circles); the room contained infected, preclinical animals (upwards triangles); the room contained clinically affected animals (downward triangles); the room was being cleaned after contamination (squares); or the room was clean and empty (diamonds). Download FIG S6, TIF file, 0.4 MB. Copyright © 2020 Colenutt et al. 2020 Colenutt et al. This content is distributed under the terms of the Creative Commons Attribution 4.0 International license.

## DISCUSSION

In this study, we have demonstrated that a contaminated environment can serve as an effective source for onward transmission of FMDV and quantified the relationship between the level of contamination and transmission risk. Indirect transmission of FMDV via contaminated surfaces or spaces has been documented previously in both experimental work (6) and anecdotal reports from outbreaks (17–19). This presents a comparable situation to other diseases, which also have an environmental transmission component that forms a minor but relevant part of the epidemiology (bTB, avian influenza, noroviruses).

The estimated *R*_0_ for environmental transmission of FMDV in this study is 1.65, which is similar to the findings of a previous study (*R*_0_ = 1.9; 95% confidence interval: 1.0 to 3.8) (6). The *R*_0_ for environmental transmission is much lower than that for direct animal-to-animal transmission, for which estimates are around 10 to 20 (20–23). However, with an *R*_0_ greater than 1, environmental transmission alone would be sufficient to sustain an outbreak if appropriate control measures were not imposed. The 2001 outbreak of FMDV in the United Kingdom demonstrated how new cases continued to occur even after the introduction of control measures, such as the culling of infected animals, movement restrictions, and biosecurity regulations (9). Additional control measures, such as use of vaccination, would also aid in reducing spread as viral shedding and susceptibility of livestock would both be reduced (24). Continued transmission when direct contact between animals is prevented demonstrates that other routes of transmission, such as indirect contact via contaminated environments and movement of fomites, must play a role in maintaining an outbreak.

**FIG 5:**
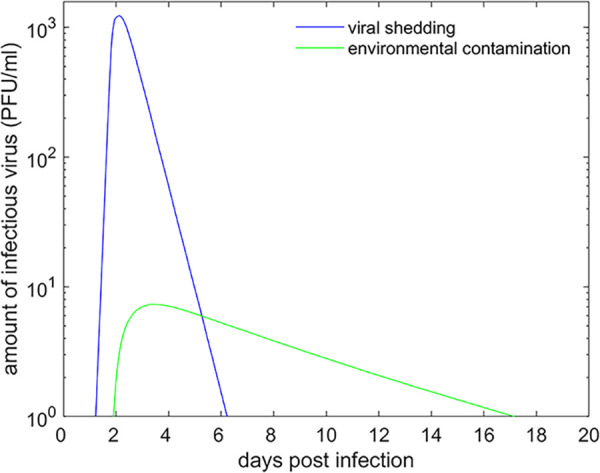
Comparison of predicted viral shedding (blue line) and environmental contamination (green line) for an individual bovine infected with foot-and-mouth disease virus. The figure shows the posterior median for the level of viral shedding, computed using equation 1 (see the Materials and Methods section), and the posterior median for the level of environmental contamination, computed using equation 2.

The difference in *R*_0_ between direct contact and environmental transmission reflects the considerably higher titers of virus in secretions and excretions from infected animals compared with those recovered from a contaminated environment (Fig. 5). Similarly, the dose response parameter (*β*) was lower than that calculated in previous direct-contact transmission studies (25). This reflects the differences between direct and environmental transmission, as lower viral titers and longer exposure periods are associated with environmental transmission. Additionally, when in direct contact, virus can be readily transmitted in contaminated aerosols generated by infected individuals. Once virus is deposited in an environment, it must be resuspended before transmission can take place. The period of potential infectivity associated with an infected individual is typically 4 to 5 days but, in contrast, based on estimated decay rates, the environment has the potential to sustain infectivity for up to 14 days (Fig. 5). These results align with the transmission pattern for FMDV, with direct contact being the primary mode of transmission and indirect transmission as a secondary route. The duration of infectiousness associated with environmental contamination, even at a lower level than in clinical secretions, provides additional risk of onward transmission once infected animals have been removed from an environment. The environmental transmission component creates a more complex scenario when assessing the risk of transmission from a case of FMDV, as has been observed with other viruses that are able to maintain infectivity within the environment (3, 26).

The EU Directive on “Community measures for the control of FMD” (27) stipulates a 21-day minimum waiting period after final cleansing and disinfection before restocking can occur, which is based on past experience of the recrudescence of FMD. As delays in restocking of farms and restrictions on the use of animal markets will contribute to the economic impact of an outbreak, imposed restrictions and waiting periods should be well justified in terms of reducing risk. Our results demonstrate that contaminated environments have the potential to remain infectious for up to 14 days and others have reported the persistence of viable FMDV in environments for weeks or even months (12, 13, 28). Survival of FMDV within environments will be variable, as demonstrated by the variation in virus half-life on different surfaces, as multiple ambient and microclimatic factors contribute to the inactivation of viruses in an environment, including temperature, relative humidity, pH, and the strain of FMDV involved (14, 29, 30). Conversely, the presence of organic material in an environment has been noted to improve the stability of FMDV (14). The survival of FMDV reported in this study was investigated using particular temperature and relative humidity (RH) conditions (RH > 60% and temperature between 18°C and 20°C). Not all environments where virus is shed in excretions and secretions or deposited will support the survival of virus. However, it should be noted that even where the general conditions would not support the continued viability of virions, places could exist where local conditions do support survival, for example, in cattle sheds, transport vehicles, or animal housing. If cleaning and decontamination is carried out effectively and thoroughly (31), then the risk of transmission can be substantially reduced, but not completely eliminated (Fig. 4). Therefore, although the risk of transmission through the environment will reduce over time and with appropriate decontamination procedures (Fig. 4), in view of the impact of outbreaks in FMDV-free countries (7), the results of this study support the current regulations for repopulation of previously contaminated spaces. In addition, we have also demonstrated that the presence of virus can be detected in environmental samples, suggesting that methods for environmental sampling could be used to measure the efficacy of decontamination procedures.

Successful environmental challenges demonstrate that risk of transmission is not only linked to contamination of the environment by individuals with clinically apparent infection (Table 1). Rooms that were contaminated with preclinical emissions from FMDV-infected cattle also produced transmission events. This highlights an important consideration in controlling outbreaks, as individuals may shed virus before clinical signs are evident. Any spaces and locations occupied by animals prior to development of clinical signs could become contaminated, though the level of contamination will depend on how long the infected animal was present and the extent of viral shedding. This study addresses viral shedding and contamination from infected cattle, but it is worth noting that the levels of viral shedding and susceptibility to infection differ among livestock species (8, 32, 33). Environmental contamination and the survival of FMDV will therefore present a risk to all livestock species, albeit at different sensitivities. In this context, the need for contact tracing is reinforced to facilitate appropriate decontamination procedures for all at-risk spaces and locations, as well as identifying at-risk individuals.

The experimental design in this study was carefully considered to prevent the possibility of unintended transmission events. Biosecurity protocols applied to staff and equipment were implemented to ensure no transfer of virus between experimental rooms occurred. Where movement of cattle was necessary, appropriate disinfection of movement corridors was carried out and a settling period observed before any further movements were made. In addition to the experimental protocol, the timings of infection and virus detection are consistent with the assumed routes of transmission (Fig. S1 in the supplemental material). Furthermore, sequence data generated from probang samples collected during experiment 2 were used to demonstrate a transmission chain that is consistent with the planned order of transmission (Fig. S2). These samples were not collected with this analysis in mind, and as such do not represent the optimum sample type for such an analysis (34). The resulting phylogenetic tree does, however, provide support for the environmental challenges being the source of transmission.

In this study, “environment” refers to the experimental room where inoculated or contact-infected calves were housed and shed virus. In the context of applying our findings to an outbreak situation, the environment could be any location where an infected animal has shed virus that has been deposited onto local surfaces (e.g., feed troughs, bedding, flooring/ground, walls). The most likely route of transmission is through inhalation of reaerosolized virus from contaminated surfaces in the experimental rooms, as cattle are more susceptible to infection through inhalation than ingestion (8). Use of aerosol sampling throughout the study demonstrated the presence of FMDV in collected aerosols, including those taken during the environmental challenges (Fig. S6) where FMDV present in aerosols would have been from the environment, as only naive cattle were present. Resuspension of FMDV provides potential for both inhalation by cattle but also relocation to new surfaces within the room. Not all areas in which virus can be deposited will be relevant to the transmission of virus and subsequent control efforts. However, our data demonstrate how easily the surroundings of an infected individual can become contaminated.

In conclusion, data from this study demonstrate the relationship between environmental contamination with FMDV and the risk of transmission. Viral persistence outside the host extends the period of infectiousness where transmission may occur, even after culling and removal of infected hosts. Some risk of transmission can remain even at low levels of contamination, and survival estimates for FMDV in the environment support the requirement for strict decontamination and waiting periods before contaminated premises can be restocked with susceptible species. A key observation of this study was the detection of contamination in environments before the appearance of clinical signs in infected animals. This highlights the importance of tracing animal movements in order to facilitate decontamination of all potentially contaminated spaces, as well as minimizing movement of virus by fomites. The observations from this study can be applied to other pathogens that are capable of survival in the environment outside the host. Awareness of this aspect of the biology of a pathogen is critical in fully understanding transmission risks.

## MATERIALS AND METHODS

### Ethics statement.

All animal experiments were carried out in accordance with the UK Animal Scientific Procedure Act (ASPA) 1986 and with the Dutch Animal Ethics law, which transpose European Directive 2010/63/EU into national law. The animal studies were approved by the UK Home Office in granting project license 70/7253 under the ASPA and all protocols underwent appropriate local ethical review procedures by both the Animal Welfare and Ethics Review Board of the Pirbright Institute and by the animal experiment committee at Wageningen BioVeterinary Research.

### Animal experiments.

**FIG 6:**
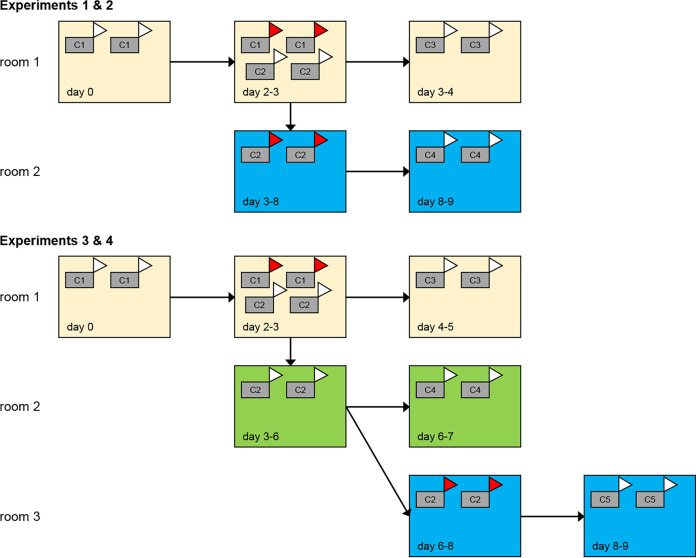
Schematic diagram showing the design of the environmental transmission experiments for foot-and-mouth disease virus in cattle. The days indicate approximate timings when the room was contaminated or when challenges took place in the room. C1 denotes needle-inoculated cattle, C2 denotes contact-challenged cattle, and C3 to C5 denote cattle challenged by exposure to an environment contaminated by two needle-inoculated animals (magnolia), two contact-challenged animals showing clinical signs (azure), or two contact-challenged animals prior to the onset of clinical signs (emerald). Donor cattle used to contaminate the room showing clinical signs of foot-and-mouth disease while in a room are shown in red.

The experiments took place in high-containment animal facilities. Experiments 1 and 2 were carried out at Wageningen BioVeterinary Research, while experiments 3 and 4 were carried out at The Pirbright Institute. Experiments 2 and 4 were direct repeats of experiments 1 and 3, respectively. Considerations for both animal welfare and biosecurity were the same between experiments. All staff and animal movements were planned to avoid unintentional movement of or exposure to FMDV. Experiments 1 and 2 followed the same formats, while minor alterations were made in experiments 3 and 4 to include an extra exposure scenario (Fig. 6). For all experiments, relative humidity in cattle housing was kept above 60% and the temperature between 18°C and 20°C. Cattle were fed daily and water was available *ad libitum* throughout the experiment. Blood, nasal swabs, oral swabs, and either throat swabs (experiments 1 and 2) or probang (oropharyngeal scraping) samples (experiments 3 and 4) were collected daily from cattle once they had been challenged. Additionally, probang samples were collected every third day during experiments 1 and 2. All animals in the study were examined twice daily, with rectal temperature and clinical signs scored and recorded once per day. Clinical scores were assigned to individual cattle based on the appearance of clinical signs, with a point awarded for each of the following: nasal secretions/drooling, lesion on mouth area, lesion on nose area, lesion on foot (1 point per foot). Provisions for humane endpoints were in place, but not reached. Cattle were culled at predetermined points (C1 and C2 pairs), or at the point of first detection of vesicular lesions on either the foot or mouth area (C3, 4, and 5 pairs).

### Experiments 1 and 2.

Initial infection of the primary pair of cattle (C1) was carried out by needle inoculation with FMDV O UKG/34/2001. Specifically, 0.2 ml of 1 × 10^5^ TCID_50_ (50% tissue culture infective dose) challenge virus was administered by intradermolingual route, 0.1 ml at each of two sites. Challenge was carried out while cattle were under sedation after administration of xylazine (0.1 to 0.2 mg per kg). When C1 donors began to show signs of FMD (2 days post infection [dpi]), a second pair of cattle (C2) was placed into the room with the C1 pair for 24 h to facilitate a direct-contact challenge. The C1 pair were then euthanized and the C2 pair moved to a second room (3 dpi). A further pair of cattle (C3) was then introduced to room 1, which had housed the C1 and C2 pairs. After a 24-h challenge period, the C3 pair was moved to a clean room and observed for onset of clinical signs. C2 cattle were kept in room 2 for 3 days after clinical signs were apparent. At this point (8 dpi), C2 cattle were removed from the study and euthanized. A final pair of cattle (C4) was then introduced to room 2 and housed there for 24 h. After the challenge period, the C4 pair was moved to a clean room and observed for onset of clinical signs. Once clinical signs were observed and confirmed in the C3 or C4 cattle, the pair was removed from the study and euthanized. Individual calves were not kept by themselves on welfare grounds, so if one of the pair developed clinical signs before the other, both individuals were euthanized. In addition, if the second calf in the pair subsequently developed clinical disease, it would be difficult to determine whether it had become infected via the environmental challenge or via direct contact from the other calf. Rooms used for environmental challenges underwent minimal maintenance, including replenishment of feed and water and removal of excess feces, but were not cleaned while housing infected or challenge pairs.

### Experiments 3 and 4.

The same format was followed for these two experiments as described for experiments 1 and 2 above, with the following alterations made to the protocol (Fig. 6). For the exposure of the C3 pair, a 24-h period was observed before the introduction of the C3 pair to room 1. To create an additional challenge scenario in these experiments, C2 cattle were observed and moved to a third room at the onset of clinical signs. The C2 pair was then kept in room 3 for 3 days once clinical signs had developed. This resulted in having two contaminated rooms, one with preclinical FMDV emissions (room 2) and one with emissions from clinically affected cattle (room 3). Room 2 had an exposure pair (C4) introduced after the C2 pair was moved to room 3, and housed the C4 pair for a 24-h exposure period. The C2 pair was removed from room 3 and euthanized after 3 days of observed clinical signs. At this point, a final pair of cattle (C5) was introduced to room 3 for a 24-h exposure period. After the respective exposure periods, the C4 and C5 pairs were moved to clean rooms and observed for the onset of clinical signs.

### Environmental sampling.

Floor (*n* = 5), wall (*n* = 5), and feed trough (*n* = 2) swabs were collected daily from animal rooms. Swabs used were nonscented electrostatic dust cloths (Minky, UK) and were added directly to 10 ml of medium (GMEM [Gibco, UK] with 1% antibiotics [penicillin-streptomycin; Gibco, UK]) after swabbing a specific area. The area size of surface swabbed was kept consistent between surfaces and sampling occasions (approximately 10 cm^2^ area of each surface). Swabs were fully saturated in medium, then vortexed briefly. A disposable wooden spatula was used to remove the cloth, at the same time pressing it to extract as much medium as possible. Aliquots of media were collected and stored at –80°C until analysis could be carried out. Fecal samples were collected from the floor of animal rooms rather than directly from animals. Fecal suspensions were made by adding 1 g of feces to 10 ml of medium (GMEM [Gibco, UK] with 1% antibiotics [penicillin-streptomycin; Gibco, UK]), vortexing, and then leaving in suspension for 30 min. Suspensions were then centrifuged (3,000 × *g* for 10 min at 4°C) to remove solid material and aliquots were made from the supernatant.

Environmental samples were collected daily in rooms occupied by animals to assess levels of environmental contamination. For the virus survival component of the study, environmental samples were collected daily for 7 days after cattle had vacated the contaminated rooms.

### Air sampling.

Aerosol samples were collected using the Coriolis μ (Bertin Technologies). The sampler was run for 10 min with an airflow rate of 300 liters/min. Samples were either collected in recently vacated rooms or in close proximity to cattle. Impinger fluid (GMEM [Gibco, UK] with 1% antibiotics [penicillin-streptomycin and amphotericin-B; Gibco, UK]), 5% BSA [Sigma-Aldrich, UK], and 1 M HEPES [Gibco, UK]) was used as collection medium. After samples had been collected, aliquots were made and stored at –80°C until analysis was carried out.

### Sample processing.

RNA was extracted from samples using the MagMAX-96 viral RNA isolation kit (Thermo Fisher Scientific) on the KingFisher Flex automated extraction platform (Thermo Fisher Scientific). Sample (50 μl) was added to 130 μl of lysis buffer (MagMAX-96 viral RNA isolation kit, Thermo Fisher Scientific) and then the manufacturer’s protocol for extraction was followed. Final elution volume for RNA was 90 μl. RNA was analyzed by reverse transcriptase PCR (rRT-PCR) on the ABI 7500 (Applied Biosystems) using the previously described Callahan protocol (35). No concentration or pooling of samples was used. A standard curve of the challenge virus was used to produce equivalent TCID_50_/ml titers for each sample.

Plaque assays were carried out using a fetal goat tongue cell line (ZZ-R 127) (36). Freshly prepared monolayers of cells in 6-well plates were infected with 200 μl of sample, overlaid with indubiose and incubated for 48 h. Serial dilutions of samples were used where necessary and each sample was tested in duplicate. No concentration or pooling of samples was carried out. Virus was inactivated using citric acid, then overlay removed and cells stained using naphthol blue. Plaque counts were made and recorded by visual inspection of plates. Challenge virus was included as a positive control.

### Sequencing and transmission chains.

Probang samples collected during experiment 2 were used to construct a transmission chain for that experiment. Experiment 2 was the only study in which sequences were generated from all probang samples, so was the only one for which a transmission chain could be generated. Viral RNA was extracted from probang samples using the RNeasy minikit (Qiagen Ltd., UK), according to the manufacturer’s protocol. The viral genomes were sequenced using MiSeq technology (Illumina, USA), as previously described (37). Assembly of raw paired-end reads to consensus-level sequences was undertaken using SeqMan NGen and SeqMan Pro (Lasergene package version 12; DNAStar, Inc., Madison, WI). The mean coverage for all newly generated sequences was 1.4 × 10^3^ and ranged from 1.3 × 10^1^ to 9 × 10^4^. All whole-genome sequences were trimmed for phylogenetic analyses to a length of 8,183 bp. Statistical parsimony network analyses were performed using the tempnet package (38) in R (version 3.6.0) (39).

### Quantifying environmental transmission.

The model used to quantify environmental transmission has three linked components. The first describes virus shedding by needle-inoculated and contact-infected animals (i.e., those which contaminated the environments in the transmission experiments). The second describes the dynamics of virus in the environment (i.e., contamination and virus survival). The third describes the probability of transmission following exposure to a contaminated environment (i.e., the dose-response relationship).

### Virus shedding.

For an acute viral infection such as FMDV, viral titers (assumed to be proportional to the level of shedding by an individual) typically rise exponentially after infection, reaching a maximum level after which they decay exponentially as the immune response clears the virus (40). This pattern can be captured by a simple phenomenological model, which also reflects the within-host dynamics of infection (40, 41). In this case, the level of viral shedding (PFU/ml) by an animal at *τ* days postinfection is given by,

(1) $$V(\tau )=\frac{2V_{p}}{\text{exp}(-\lambda_{g}(\tau -T_{p}))+\text{exp}(\lambda_{d}(\tau -T_{p}))}$$

where *V_p_* is the level of peak virus shedding, *T_p_* is the time of peak shedding, and *λ_g_* and *λ_d_* are the rates for the exponential growth and decay phases, respectively. Individual variation in shedding is incorporated by allowing each of the parameters (i.e., *V_p_*, *T_p_*, *λ_g_*_,_ and *λ_d_*) to vary among individuals. In this study, the total amount of virus isolated from nasal and oral swabs for an animal was used as a proxy measure for total virus shedding.

### Environmental contamination and virus survival.

The level of virus (PFU/ml) in environmental samples (i.e., feces or swabs taken from the floor, walls, or feed trough) was assumed to vary according to the amount of virus shed by infected animals and the rate at which virus decays in the sample. In this case, the mean level of virus in sample type *j* is described by the following differential equation,

(2) $$\frac{\text{d}E_{j}}{\text{d}t}=\alpha_{j}\sum_{i}V_{i}(t)-\delta_{j}E_{j}(t)$$

where *V_i_*(*t*) is the level of virus shedding in the room by animal *i* at time *t* (see equation 1), summed over all animals in the room; *α_j_* is the rate of contamination; and *δ_j_* is the rate of decay of virus in the sample.

### Dose-response relationship for environmental transmission.

The probability of transmission (i.e., that an animal would be infected and show clinical signs) following exposure to a contaminated environment was assumed to depend on the level of virus (PFU/ml) in the environment and the duration of exposure. Specifically, an exponential dose-response model (42) was assumed, so that the probability is given by,

(3) $$p=1-\text{exp}\left(-\beta \sum_{j}\int_{t_{C}}^{t_{C}+1}E_{j}(t)\text{d}t\right)$$

where *β* is the transmission rate, *E_j_* is the mean level of virus in sample type *j* (given by equation 2) and *t*_C_ is the time of first exposure.

### Parameter estimation.

Parameters in the model described by equations 1 to 3 were estimated by fitting it to data on virus isolation from samples taken from the needle-inoculated and contact-challenged cattle (nasal and oral swabs, with the total quantity of virus from both taken as a proxy for overall shedding by an infected animal), on virus isolation from the environmental samples, and on the outcome of each environmental challenge. Parameters were estimated in a Bayesian framework, full details of which are presented in Text S1.

10.1128/mBio.00381-20.10 TEXT S1 Bayesian inference of parameters quantifying environmental transmission of foot-and-mouth disease virus. Download Text S1, DOCX file, 0.1 MB. Copyright © 2020 Colenutt et al. 2020 Colenutt et al. This content is distributed under the terms of the Creative Commons Attribution 4.0 International license.

### Basic reproduction number for environmental transmission.

For the model of environmental transmission described by equations 1 to 3, the reproduction number *R*_0_ is given by,

(4) $$R_{0}=\beta \int_{0}^{\infty }E(t)\text{d}t$$

where *E*(*t*) is the mean level of viral contamination for a single animal (computed using equations 1 and 2; see Text S1 for full details).

As a comparison, a second method, based on the attack rate (i.e., the proportion of exposures resulting in successful transmission), was also used to calculate *R*_0_ for environmental transmission using the R0 package (43) in R (version 3.6.0) (39).

### Estimating viral RNA decay rates.

Decay rates for FMDV RNA in different sample types (floor, wall, feed trough, or feces) were estimated by fitting exponential decay curves to data quantifying levels of viral RNA in each sample type (see Text S1 for details).

10.1128/mBio.00381-20.1 DATA SET S1 Levels of infectious virus and viral RNA in samples taken from calves, ambient air, and the environment during experiments to quantify environmental transmission of foot-and-mouth disease virus. Download Data Set S1, XLSX file, 0.2 MB. Copyright © 2020 Colenutt et al. 2020 Colenutt et al. This content is distributed under the terms of the Creative Commons Attribution 4.0 International license.
