# Supplementary figures and images for: Quantifying the Transmission of Foot-and-Mouth Disease Virus in Cattle via a Contaminated Environment
Source: mBio. 2020 Aug 4;11(4):e00381-20. doi: 10.1128/mBio.00381-20 (PMC7407078; doi:10.1128/mBio.00381-20)

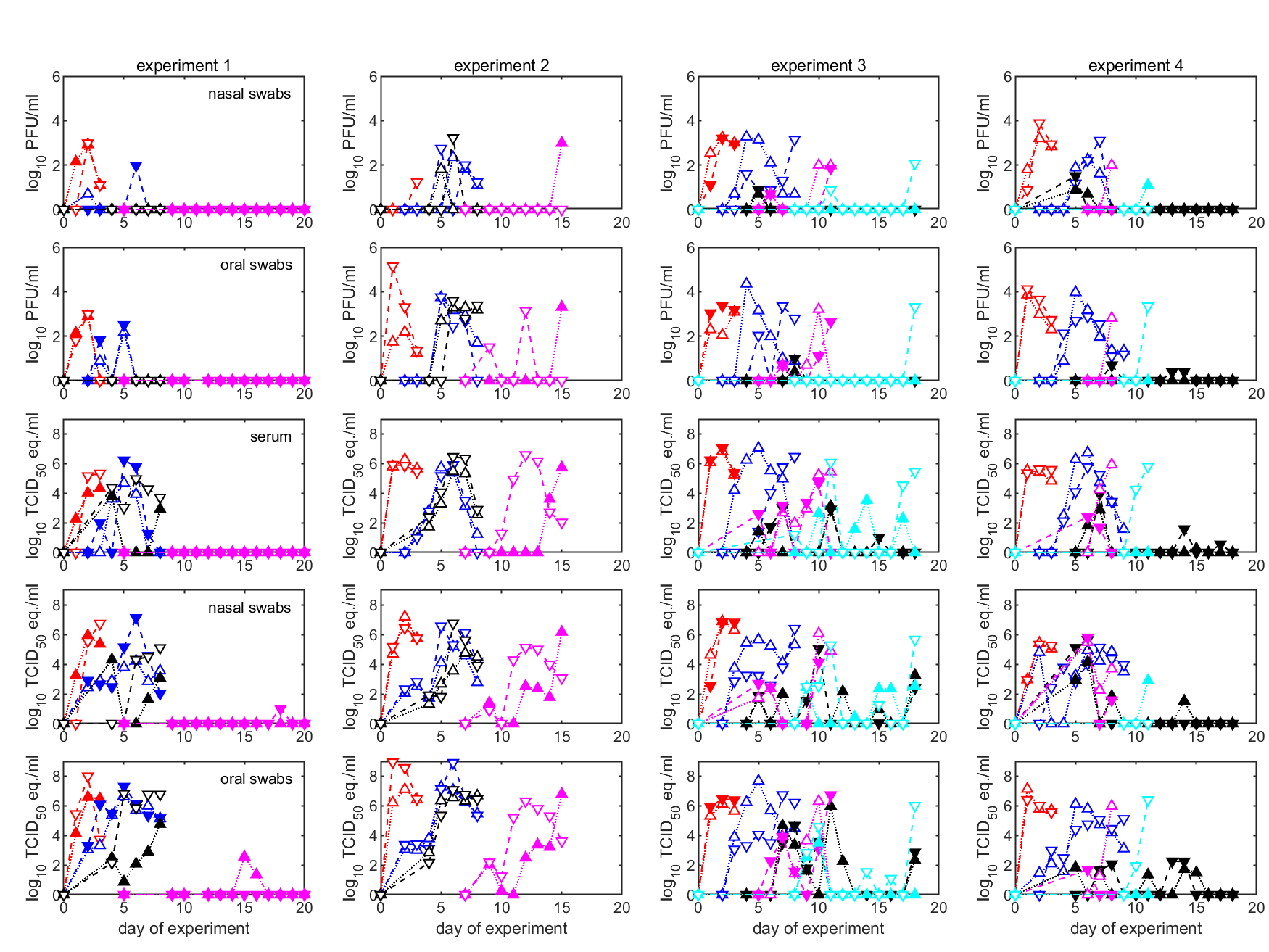

Supplement: FIG S1 [file mBio.00381-20-sf001.tif]

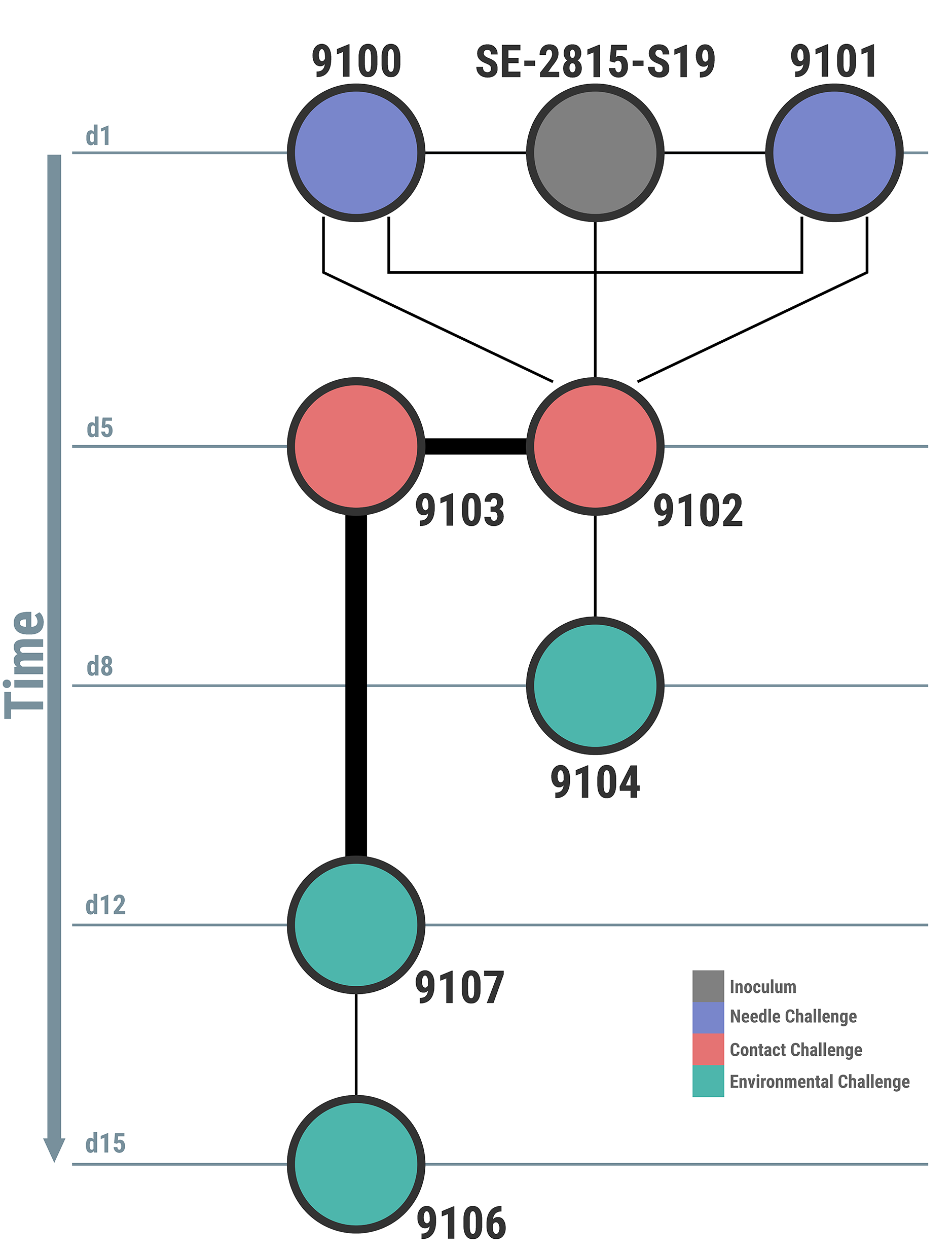

Supplement: FIG S2 [file mBio.00381-20-sf002.tif]

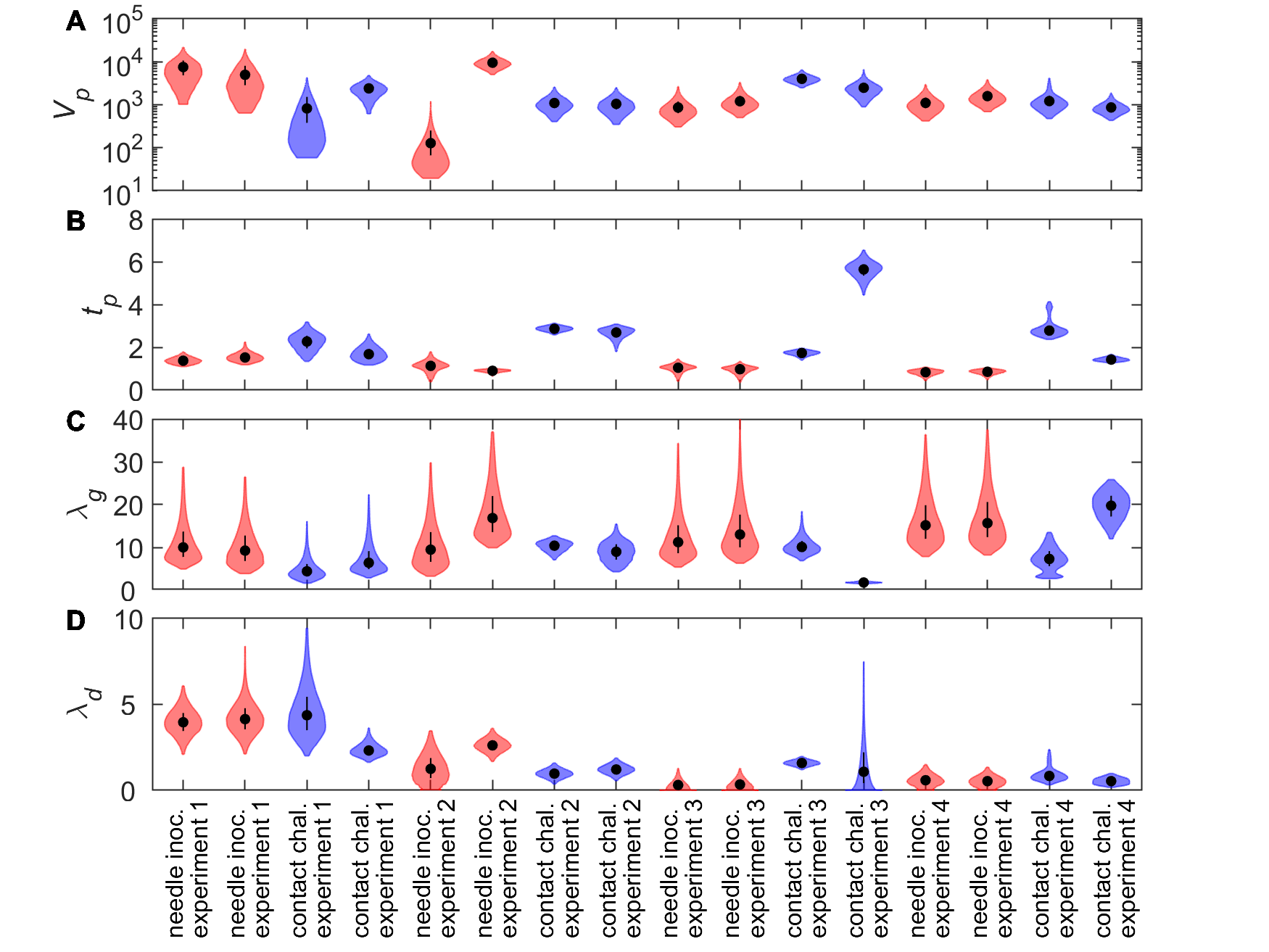

Supplement: FIG S3 [file mBio.00381-20-sf003.tif]

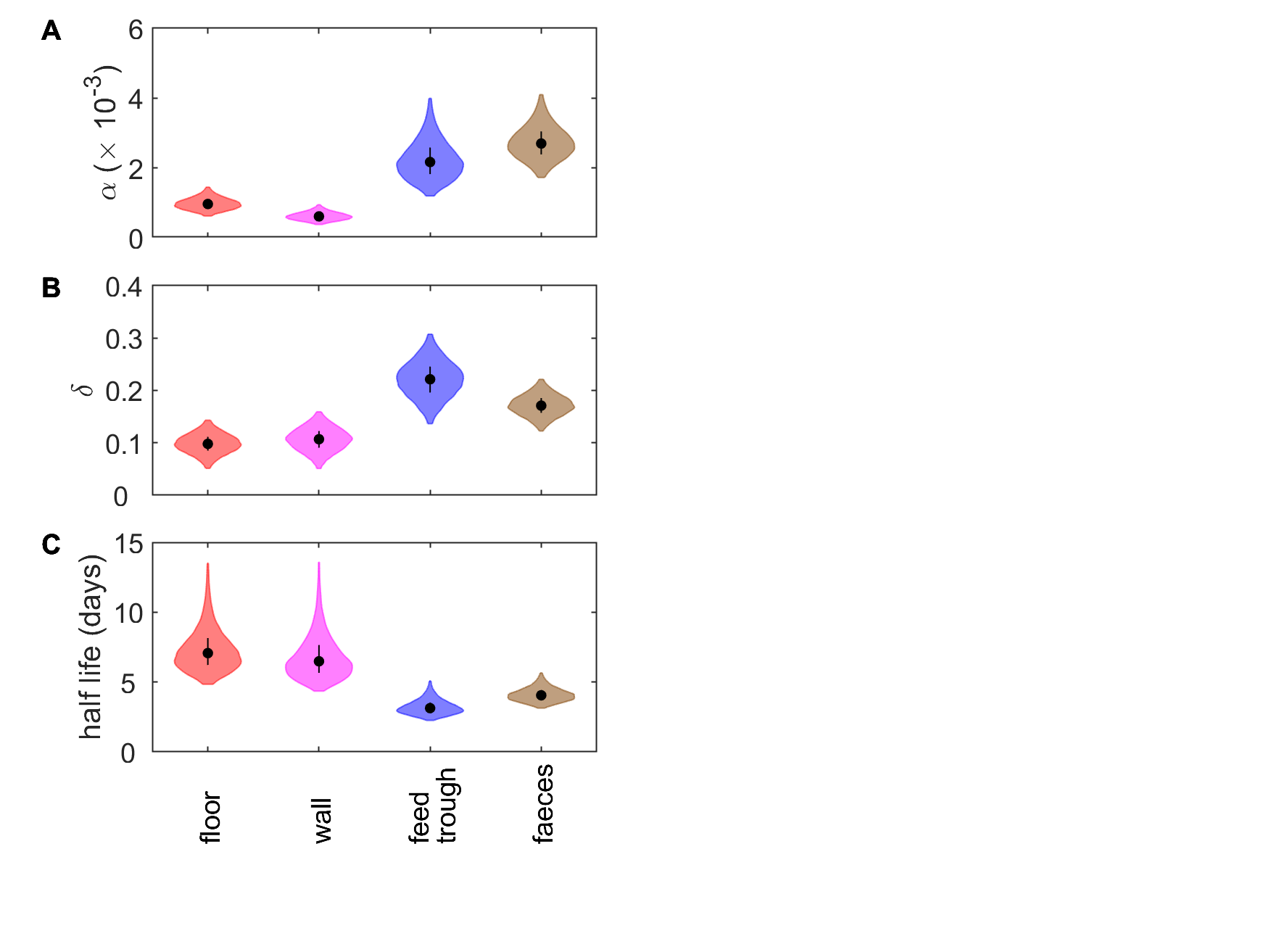

Supplement: FIG S4 [file mBio.00381-20-sf004.tif]

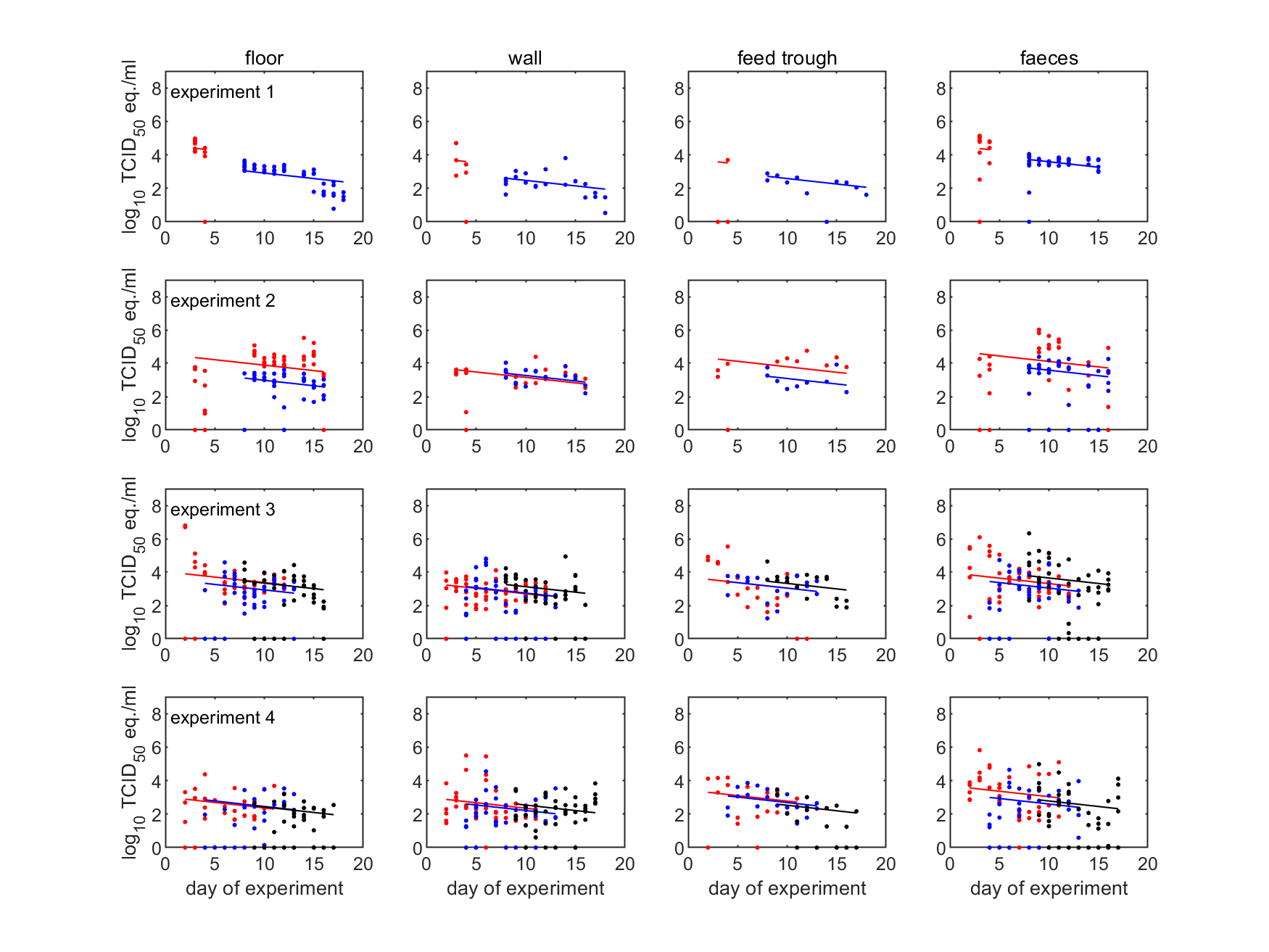

Supplement: FIG S5 [file mBio.00381-20-sf005.tif]

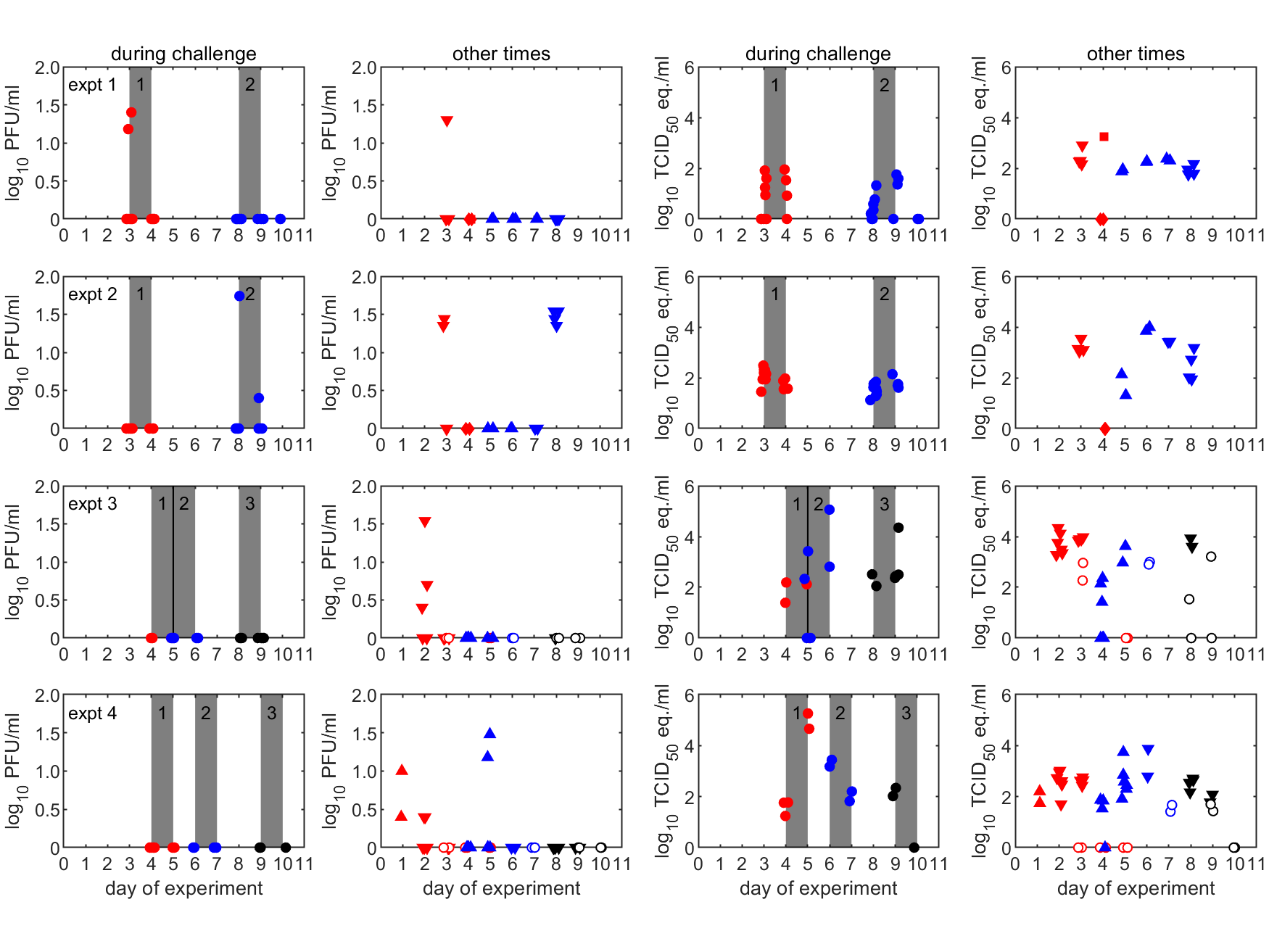

Supplement: FIG S6 [file mBio.00381-20-sf006.tif]
