# Supplementary material for: Quantifying the Transmission of Foot-and-Mouth Disease Virus in Cattle via a Contaminated Environment
Source: mBio. 2020 Aug 4;11(4):e00381-20. doi: 10.1128/mBio.00381-20 (PMC7407078; doi:10.1128/mBio.00381-20)
Supplement: TABLE S1 [file mBio.00381-20-st001.docx]

**Table S1.** Comparison of contamination and decay rates for foot-and-mouth disease virus amongst sample types.

| model | deviance information criterion |
| --- | --- |
| *infectious virus* |  |
| contamination and decay rates common | 3065 |
| contamination rates common, decay rates vary | 2999 |
| contamination rates vary, decay rate common | 2936 |
| contamination and decay rates vary | 2921 |
| *viral RNA* |  |
| decay rate common | 2698 |
| decay rates vary | 2770 |
