# Supplementary material for: Quantifying the Transmission of Foot-and-Mouth Disease Virus in Cattle via a Contaminated Environment
Source: mBio. 2020 Aug 4;11(4):e00381-20. doi: 10.1128/mBio.00381-20 (PMC7407078; doi:10.1128/mBio.00381-20)
Supplement: TABLE S2 [file mBio.00381-20-st002.docx]

**Table S2.** Estimates for environmental transmission parameters for foot-and-mouth disease virus.

| parameter | symbol | posterior median | 95% credible limits | |
| --- | --- | --- | --- | --- |
|  |  |  | lower | upper |
| *infectious virus* |  |  |  |  |
| rate of contamination (day^-1^) | *α* |  |  |  |
| floor swab |  | 9.7 × 10^-4^ | 6.6 × 10^-4^ | 1.4 × 10^-3^ |
| wall swab |  | 6.1 × 10^-4^ | 4.1 × 10^-4^ | 8.9 × 10^-4^ |
| feed trough |  | 2.2 × 10^-3^ | 1.3 × 10^-3^ | 3.6 × 10^-3^ |
| faeces |  | 2.7 × 10^-3^ | 1.9 × 10^-3^ | 3.8 × 10^-3^ |
| decay rate (day^-1^) | *δ* |  |  |  |
| floor swab |  | 0.098 | 0.059 | 0.14 |
| wall swab |  | 0.11 | 0.060 | 0.15 |
| feed trough |  | 0.22 | 0.15 | 0.29 |
| faeces |  | 0.17 | 0.13 | 0.21 |
| half life (days) |  |  |  |  |
| floor swab |  | 7.1 | 5.1 | 11.8 |
| wall swab |  | 6.5 | 4.6 | 11.5 |
| feed trough |  | 3.2 | 2.4 | 4.6 |
| faeces |  | 4.1 | 3.3 | 5.3 |
| transmission rate (pfu^-1^) | *β* | 0.027 | 0.011 | 0.057 |
| basic reproduction number | *R*_0_ |  |  |  |
| transmission model* |  | 1.7 | 0.5 | 4.5 |
| attack rate* |  | 1.7 | 1.3 | 4.2 |
| *viral RNA* |  |  |  |  |
| decay rate (day^-1^) |  | 0.065 | 0.050 | 0.081 |
| half life (days) |  | 10.6 | 8.5 | 13.8 |

* method of estimation
