# Supplementary material for: Quantifying the Transmission of Foot-and-Mouth Disease Virus in Cattle via a Contaminated Environment
Source: mBio. 2020 Aug 4;11(4):e00381-20. doi: 10.1128/mBio.00381-20 (PMC7407078; doi:10.1128/mBio.00381-20)
Supplement: TEXT S1 [file mBio.00381-20-s0001.docx]

**Text S1. Bayesian inference of parameters quantifying environmental transmission of foot-and-mouth disease virus**

Here we provide full details of the model used to quantify environmental transmission of foot-and-mouth disease virus (FMDV) in cattle, including the Bayesian framework used to estimate parameters in the model.

***S1.1 Virus shedding***

Virus shedding curves were inferred for needle-inoculated and contact-infected cattle (i.e. those which contaminated the environments used in the transmission experiments) by fitting to data on total amount of virus isolated from nasal and oral swabs (used as a proxy measure for total virus shedding). A simple phenomenological model was used in which viral titre rises exponentially after infection, reaching a maximum level after which it decays exponentially [1,2]. The level of virus shedding by animal *i* at *τ* days post infection is given by,

$$V_{i}(\tau)=\frac{2V_{p}^{(i)}}{\exp(-\lambda_{g}^{(i)}(\tau-T_{p}^{(i)}))+\exp(\lambda_{d}^{(i)}(\tau-T_{p}^{(i)}))},$$

where $V_{p}^{(i)}$ is the level of peak virus shedding, $T_{p}^{(i)}$ is the time of peak shedding and $\lambda_{g}^{(i)}$ and $\lambda_{d}^{(i)}$ are the rates during the exponential growth and decay phases, respectively, for the animal.

Individual variation in shedding is incorporated by allowing each of the parameters (i.e. *V_p_*, *T_p_*, *λ_g_* and *λ_d_*) to vary amongst individuals, such that they are drawn from higher-order gamma distributions (i.e. there is hierarchical structure in the parameters). In this case, the parameters for animal *i* are given by,

$$\begin{matrix} V_{p}^{(i)}\sim\text{Gamma}(s_{V},\mu_{V}), \\ T_{p}^{(i)}\sim\text{Gamma}(s_{T},\mu_{T}), \\ \lambda_{g}^{(i)}\sim\text{Gamma}(s_{\lambda_{g}},\mu_{\lambda_{g}}), \\ \lambda_{d}^{(i)}\sim\text{Gamma}(s_{\lambda_{d}},\mu_{\lambda_{d}}), \end{matrix}$$

where *s* and *μ* are the shape parameter and mean of the gamma distributions, respectively.

***S1.2 Environmental contamination and virus survival***

The level of virus in environmental samples (i.e. faeces or swabs taken from the floor, walls or food trough) was assumed to vary according to the amount of virus shed by infected animals and the rate at which virus decays in the sample. In this case, the mean level of virus in sample type *j* from room *r* is described by the following ordinary differential equation (ODE),

$$\frac{\text{d}E_{jr}}{\text{d}t}=\alpha_{j}\sum_{i} V_{i}(t)-\delta_{j}E_{jr}(t),$$

where *V_i_*(*t*) is the level of virus shedding in the room by animal *i* at time *t* (given by equation ), summed over all animals in the room, *α_j_* is the rate of contamination and *δ_j_* is the rate of decay of virus in the sample.

Differences in rates of contamination and viral decay amongst sample types are incorporated by assuming the parameters are drawn from higher-order gamma distributions (i.e. there is hierarchical structure in the parameters). Specifically, the rates of contamination and decay for sample type *j* are given by,

$$\begin{matrix} \alpha_{j}\sim\text{Gamma}(s_{\alpha},\mu_{\alpha}), \\ \delta_{j}\sim\text{Gamma}(s_{\delta},\mu_{\delta}), \end{matrix}$$

where *s* and *μ* are the shape parameter and mean of the distributions, respectively. Four possibilities were considered for the contamination and decay rates: (i) they were common to all sample types; (ii) the contamination rate was common to all sample types and the decay rates varied amongst sample types; (iii) the contamination rates varied amongst sample types and the decay rate is common to all sample types; and (iv) the contamination and decay rates varied amongst sample types. The different models were compared using the deviance information criterion (DIC) [3].

An additional level of hierarchy in which rates of contamination and decay varied amongst rooms (as well as sample types) was also considered. However, the model failed to converge in this case, indicating problems with identifiability in the more complex parameter structure.

***S1.3 Dose-response relationship for environmental transmission***

The probability of transmission (i.e. that an animal would be infected and show clinical signs) following exposure to a contaminated environment was assumed to depend on the level of virus in the environment and the duration of exposure. Specifically, an exponential dose-response model [4] was assumed, so that the probability of transmission for a calf in room *r* is given by,

$$p_{r}=1-\exp\left(-\beta\sum_{j} \int_{t_{C}^{(r)}}^{t_{C}^{(r)}+1} E_{jr}(t) \text{d}t\right),$$

where *β* is the transmission rate, *E_jr_* is the mean level of virus in sample type *j* (see equation ) and *t_C_* is the time of first exposure .

In the transmission experiments each pair of contact exposed calves was only observed until the first animal showed clinical signs. Accordingly, the probability of a successful infection (i.e. at least one animal becoming infected and showing clinical signs) is given by,

$$q_{r}=1-(1-p_{r})^{2},$$

where *p_r_* is the probability of transmission for an individual animal (given by equation ).

Model parameterisations were explored in which transmission rates were allowed to differ amongst rooms or sample types by including hierarchical structure in the parameters (cf. section S1.2). However, these models failed to converge, indicating problems with identifiability in the more complex parameterisations. In addition, we considered a model in which viral titre was replaced by log viral titre in equation , but this provided a significantly worse fit to the data as judged by the DIC [3] (DIC=2887 for the model using titre compared with DIC=2891 for the model using log titre).

***S1.4 Parameter estimation***

Parameters were estimated in a Bayesian framework. The likelihood for the data is given by,

$$\begin{matrix} L(\mathbf{\varphi}).=.\prod_{i} \prod_{t} f(\log V_{i}^{(obs)}(t)|\log V_{i}(t),\sigma_{V})^{1-c_{it}}F(0|\log V_{i}(t),\sigma_{V})^{c_{it}}\times\\ .\prod_{r} \prod_{j} \prod_{t} f(\log E_{jr}^{(obs)}(t)|\log E_{jr}(t),\sigma_{E})^{1-c_{jrt}}F(0|\log E_{jr}(t),\sigma_{E})^{c_{jrt}}\times\\ .\prod_{r} q_{r}^{d_{r}}(1-q_{r})^{1-d_{r}}, \end{matrix}$$

where **φ** is a vector of model parameters (individual level and hierarchical) and *f* and *F* are the probability and cumulative density functions for the normal distribution, respectively, and *c* is a variable indicating whether (*c*=1) or not (*c*=0) the observation is left-censored (i.e. it is below the detection threshold, set arbitrarily at 1 pfu/ml). The first term corresponds to the virus shedding curves with observed and expected titres for animal *i*, $V_{i}^{(obs)}(t) \text{and} V_{i}(t)$(given by equation ), respectively, and error standard deviation *σ_V_*. The second term corresponds to the environmental contamination with observed and expected titres for sample *j* from room *r*, $E_{jr}^{(obs)}(t) \text{and} E_{jr}(t)$(given by equation ), respectively, and error standard deviation *σ_E_*. Finally, the third term corresponds to the outcome of the environmental exposure, where *q_r_* is the probability of a successful infection in room *r* (given by equation ) and *d_r_* is an indicator variable such that *d_r_*=0 if transmission did not occur following exposure to the room and *d_r_*=1 if it did.

Priors for the individual-level parameters used the hierarchical structures described in equations for virus shedding and for environmental contamination and survival. Non-informative priors (diffuse exponential with mean 100) were used for the highest-level parameters in each hierarchical model. A non-informative prior was used for the error standard deviation for the environmental contamination curves, but an informative gamma prior (with mean 0.25 and shape 1000) was required for the error standard deviation for the virus curves. The prior mean (0.25) was derived from exploratory analysis fitting the curve for each animal , independently to the data using maximum likelihood methods.

Samples from the joint posterior distribution were generated using an adaptive Metropolis scheme [5], modified so that the scaling factor was tuned during burn-in to ensure an acceptance rate of between 20% and 40% for more efficient sampling of the target distribution [6]. Two chains of 10,000,000 iterations were run, with the preceding 10,000,000 iterations discarded to allow for burn-in of the chain. The chains were then thinned (taking every 1,000th sample) to reduce autocorrelation amongst the samples. The methods were implemented in Matlab (version R2019b; The Mathworks, Inc.) and the code is available online at <https://github.com/SimonGubbins/EnvironmentalTransmissionOfFMDV>. Convergence of the scheme was assessed visually and by examining the Gelman-Rubin statistic implemented in the coda package [7] in R (version 3.6.0) [8].

***S1.5 Basic reproduction number***

The basic reproduction number *R*_0_ for environmental transmission is given by,

$$R_{0}=\beta\int_{0}^{\infty} \sum_{j} E_{j}(t) \text{d}t,$$

where *β* is the transmission rate and *E_j_*(*t*) is the mean level of viral contamination at time *t* in sample *j* for a single animal. The level of contamination is computed by solving the following ODE (cf. equation ),

$$\frac{\text{d}E_{j}}{\text{d}t}=\alpha_{j}V(t)-\delta_{j}E_{j}(t),$$

where *V*(*t*) is the level of virus shed at time *t* (given by equation ) and *α_j_* and *δ_j_* are the rates of contamination and decay of virus on the surface *j*, respectively.

The posterior distribution for *R*_0_ was calculated using the MCMC samples for the rates of contamination and decay for each surface and for the transmission parameter (i.e. *α_j_*, *δ_j_* and *β*) and the hierarchal gamma means (see equation ) for the virus shedding parameters (i.e. *V_p_*, *t_p_*, *λ_g_* and *λ_d_*).

***S1.6 Estimating viral RNA decay rates***

Decay rates for FMDV RNA were estimated by fitting exponential decay curves to data quantifying levels of viral RNA in different sample types (floor, wall, feed trough or faeces) for each room and experiment. Two models were considered. In the first model, the decay rate was assumed to be common to all sample types, so that the expected quantity of viral RNA (in tissue culture ID_50_ equivalents/ml) at time *t* for sample type *k* in room *j* during experiment *i* is given by,

$$\log\mu_{ijk}(t)=V_{ijk}^{(0)}+bt,$$

where $V_{ijk}^{(0)}$ is the initial level of RNA and *b* is the (common) decay rate. In the second model, the decay rates were assumed to differ amongst sample types, so that,

$$\log\mu_{ijk}(t)=V_{ijk}^{(0)}+b_{k}t,$$

where *b_k_* is the decay rate for sample type *k*. Variation between sample types was incorporated assuming hierarchical structure in the decay rate, that is,

$$b_{k}\sim\text{N}(\mu_{b},\sigma_{b}^{2}).$$

Variation in initial RNA levels was incorporated in both models by assuming hierarchical structure in the parameters, so that,

$$V_{ijk}^{(0)}\sim N(\mu_{V_{0}},\sigma_{V_{0}}^{2}).$$

Parameters were estimated in a Bayesian framework. A normal likelihood was used for the data with expected values given by equations or and error variance, $\sigma_{e}^{2},$with left-censoring of those observations below the detection threshold (arbitrarily set at 1 TCID_50_ eq./ml). Priors for the initial levels and decay rates were given by the higher-order distributions, (model 2 only) and , while non-informative (diffuse Normal or diffuse exponential) priors were assumed for the higher-order parameters and the error variance. The methods were implemented in OpenBUGS (version 3.2.3; <http://openbugs.net>) and the code is available online at <https://github.com/SimonGubbins/EnvironmentalTransmissionOfFMDV>. Two chains each of 50,000 iterations were generated, with the preceding 10,000 iterations discarded to allow for burn-in of the chains. Chains were subsequently thinned (by selecting every tenth iteration) to reduce autocorrelation amongst the samples. Convergence of the chains was monitored visually and using the Gelman-Rubin statistic in OpenBUGS.

The models were compared using the DIC [3]. The lowest DIC was obtained for the model in which the decay rate was common to all surface types (DIC=2698; compared with DIC=2770 for the model in which the decay rates varied amongst surfaces).

**References for S1 Text**

1. Holder, B.P. & Beauchemin, C.A.A. 2011 Exploring the effect of biological delays in kinetic models of influenza within a host or cell culture. *BMC Public Health* **11**(Suppl 1), S10.
2. Handel, A., Lebarbenchon, C., Stallknecht, D. & Rohani, P. 2014 Trade-offs between and within scales: environmental persistence and within-host fitness of avian influenza viruses. *Proc. R. Soc. Lond. B* **281**, 20133051.
3. Spiegelhalter DJ, Best NG, Carlin BP, van der Linde A. 2002 Bayesian measures of model complexity and fit (with discussion). *J. R. Stat. Soc. B* **64**, 583-639.
4. Haas, C.N. 1983 Estimation of risk due to low doses of microorganisms: a comparison of alternative methodologies. *Amer. J. Epidemiol.* **118**, 573-582.
5. Haario, H., Saksman, E. & Tamminen, J. 2001 An adaptive Metropolis algorithm. *Bernoulli* **7**, 223-242.
6. Andrieu, C. & Thoms, J. 2008 A tutorial on adaptive MCMC. *Stat. Comput.* **18**, 343-373.
7. Plummer, M., Best, N., Cowles, K. & Vines, K. 2006 CODA: Convergence Diagnosis and Output Analysis for MCMC. *R News* **6**, 7-11.
8. R Core Team 2019 R: A language and environment for statistical computing. R Foundation for Statistical Computing, Vienna, Austria. (<http://www.R-project.org/>).
